# HCV entry receptors as potential targets for siRNA-based inhibition of HCV

**DOI:** 10.1186/1479-0556-9-15

**Published:** 2011-09-06

**Authors:** Shah Jahan, Baila Samreen, Saba Khaliq, Bushra Ijaz, Mahwish Khan, Muhammad Hassan Siddique, Waqar Ahmad, Sajida Hassan

**Affiliations:** 1 Applied and Functional Genomics Lab, Centre of Excellence in Molecular Biology, University of the Punjab, Pakistan

## Abstract

**Background:**

Hepatitis C virus (HCV) is a major health concern with almost 3% of the world's population (350 million individuals) and 10% of the Pakistani population chronically infected with this viral pathogen. The current therapy of interferon-α and ribavirin against HCV has limited efficiency, so alternative options are desperately needed. RNA interference (RNAi), which results in a sequence-specific degradation of HCV RNA has potential as a powerful alternative molecular therapeutic approach. Concerning viral entry, the HCV structural gene E2 is mainly involved in virus attachment to the host cell surface receptors i.e., CD81 tetraspanin, scavenger receptor class B type 1 (SR-B1), low density lipoprotein receptor (LDLR) and claudin1 (CLDN1).

**Results:**

In this report, we studied the relationship of the HCV receptors CD81, LDL, CLDN1 and SR-B1to HCV infection. The potential of siRNAs to inhibit HCV-3a replication in serum-infected Huh-7 cells was demonstrated by treatment with siRNAs against HCV receptors, which resulted in a significant decrease in HCV viral copy number.

**Conclusions:**

Our data clearly demonstrate that the RNAi-mediated silencing of HCV receptors is among the first of its type for the development of an effective siRNA-based therapeutic option against HCV-3a. These findings will shed further light on the possible role of receptors in inhibition of HCV-3a viral titre through siRNA mediated silencing.

## Introduction

HCV infection is a major health problem; more than 350 million people worldwide and 10% of the population in Pakistan are chronically infected with this disease [1,2]. In 40-60% of HCV-infected individuals, chronic infection is mainly associated with liver cirrhosis and steatosis, leading to hepatocellular carcinoma (HCC) [3,4]. In Pakistan, the major HCV genotype is 3a, followed by 3b and 1a, with a strong correlation between chronic HCV infection and HCC in Pakistan associated with genotype 3a [5]. About 75% of patients achieve no therapeutic benefit from the present combination therapy with pegylated interferon α (PEG-IFN-α) and ribavirin because of adverse side effects [6]. In order to get a better treatment effect, there is a desperate need to develop more efficient and better therapeutic alternatives for treating HCV infections.

The mechanism of HCV cell entry was only revealed after years of research due to the absence of suitable animal models and in vitro cell culture systems. Recently, different groups have studied HCV replication in serum-infected liver cell lines which mimics the biology of the naturally occurring HCV virions biology and the kinetics of HCV infection in humans liver cells [7-13]. HCV envelop proteins E1 and E2 are highly glycosylated and have functional roles in protein folding, HCV entry, fusion and defense against neutralization by envelope-specific antibodies [14-19]. E2 glycoproteins take part as key components in the interaction between the virus and its major cellular receptors like CD81, SR-BI and CLDN1 [20-22]. HCV enters the cell through receptors followed by the release of its viral RNA genome into the cytoplasm. CD81 is a strong candidate to serve as a HCV cell surface receptor [23-25]. HCV E2 binds with high affinity to the large extracellular loop of CD81, a tetraspanin found on the surface of different cell types, including hepatocytes and epithelial cells, and plays an important role in the early steps of viral entry [26-28]. An additional role is played by the scavenger receptor class B type I (SRBI) and low-density-lipoproteins receptor (LDLR) [29,30]. SR-BI is thought to be a putative "post binding" entry molecule of HCV [31,32]. Furthermore, interaction of HCV in association with lipoproteins and LDLR via nonspecific uptake into hepatocytes is also a possible mechanism of HCV cell entry [33,34]. Recently, the tight junction protein claudin-1 has also been identified as a late entry factor for HCV infection [35,36]. Therefore, HCV receptors are a good target to block HCV entry.

RNA interference (RNAi) is a sequence specific gene silencing mechanism induced by small interfering RNA (siRNA) to which HCV RNA is highly susceptible [37-39]. Currently, research is focused on developing this sequence-specific gene silencing for human therapy and gene function studies. Despite the limitation of sequence variability, the development of an effective RNAi-based antiviral therapy can be achieved by finding highly effective target sites and targeting HCV genes and cellular genes at the same time. Previously, we reported the development of an siRNA targeting the HCV-3a envelope proteins crucial for viral entry [40]. This method provides a better choice for development of a rational antiviral strategy against the local HCV-3a genotype.

In the present study, the inhibition of HCV entry via cellular receptors using siRNA against CD81, LDLR, SR-BI, CLDN1 was observed, which we interpreted as confirmation of the role of these receptors in mediating HCV entry. Moreover, we also showed the effect of siRNA-induced silencing of receptor genes in reducing HCV viral load in serum-infected Huh-7 cells.

## Materials and methods

### Source of samples

he local HCV 1a and HCV-3a patients' serum samples used in this investigation were obtained from the CAMB (Center for Applied Molecular Biology) diagnostic laboratory, Lahore, Pakistan after quantification and genotype determination. Serum samples were stored at -80°C prior to RNA extraction for cloning and viral inoculation experiments. Patients' written consent and approval for this study was obtained from our institutional ethical committee.

### Design and synthesis of siRNA

Design and synthesis of siRNA was done as described earlier [12,41,42]. Briefly, siRNA oligonucleotides were selected to generate RNA interference against HCV receptors using the Ambion's siRNA design tool http://www.ambion.com/techlib/misc/siRNA_finder.html. The designed siRNAs (cellular genes, HCV receptors and scrambled control) were synthesized using the Silencer siRNA construction kit according to the manufacturer's instruction (Ambion, USA).

### Viral inoculation and co-transfection with siRNA in Huh-7 cell line

The Huh-7 cell line was kindly provided by Dr. Zafar Nawaz (University of Miami, USA) and maintained in Dulbecco's modified eagle medium (DMEM) supplemented with 100 μg/ml penicillin/streptomycin and 10% fetal bovine serum (Sigma Aldrich, USA) at 37°C with 5% CO_2 _as complete medium. The medium was renewed every 3 days and cells were passaged every 4-5 days. To examine the effects of siRNAs, cells were transfected with siRNAs specific for either HCV receptors or scrambled HCV serum-infected cells.

The Huh-7 cell line was used to establish the in vitro replication of HCV-1a and 3a. A similar protocol was used for viral inoculation as described previously [11,12,42-44]. Briefly, for these experiments serum from HCV-3a patients containing a high viral titer (> 1 × 10^8 ^IU/ml) was used as principle inoculums. Huh-7 cells were maintained in 6-well culture plates to semi-confluence, washed twice with serum-free medium then inoculated with 500 μl (5 × 10^7^IU/well viral load) of HCV-3a sera and 500 μl serum-free media. Cells were maintained overnight at 37°C in 5% CO_2_. Next day, the adherent cells were washed three times with 1X PBS, complete medium was added and incubation was continued for 48 hrs. Cells were harvested and assessed for the presence of viral RNA quantitatively by real-time PCR. To analyze the effect of siRNA on HCV infection, serum infected Huh-7 cells were seeded after three days of infection in 24-well plates and grown to 80% confluence with 2 ml medium. The cells were transfected with or without 40 μM/well siRNA against cellular receptors alone or in combination using Lipofectamine™ 2000 (Invitrogen Life technologies, CA) according to the manufacturer's protocol as described earlier [12,45].

### Viral load quantification

Cells were harvested for viral load determination using the Gentra RNA isolation kit (Gentra System Pennsylvania, USA) according to the manufacturer's instructions. For viral quantification, the Sacace HCV quantitative analysis kit (Sacace Biotechnologies Caserta, Italy) was used. Briefly, 10 μl of extracted viral RNA was mixed with an internal control provided by Sacace HCV Real TM Quant kit and subjected to viral quantification using a real-time PCR SmartCycler II system (Cepheid Sunnyvale, USA).

### Total RNA isolation and gene expression analysis

Total RNA from HCV serum-infected and no infected cells was isolated using TRIzol reagent (Invitrogen life technologies, CA), 24 hrs and 48 hrs post-transfection. To analyze the effect of siRNA on envelope gene expression, cDNA was synthesized from 1 μg of total RNA using Superscript III cDNA synthesis kit (Invitrogen life technologies, CA) and semi-quantitative RT-PCR was done using primers of HCV receptors, and GAPDH as control. Quantitative real-time PCR was carried out using a Real Time ABI 7500 system (Applied Biosystems Inc, USA) with SYBR green mix (Fermentas International Inc, Canada) as described earlier [12,42,46]. The relative gene expression analysis was carried out by the SDS 3.1 software (Applied Biosystems Inc, USA). Each individual experiment was performed in triplicate.

### Western blotting

To determine the effect of siRNAs on HCV E2 protein expression levels, HCV serum-infected cells were lysed using the ProteoJET mammalian cell lysis reagent (Fermentas, Canada). Equal amounts of total proteins were subjected to electrophoresis on 12% SDS-PAGE and electrophoretically transferred to a nitrocellulose membrane according to the manufacturer's protocol (Bio-Rad, CA). After blocking nonspecific binding sites with 5% skimmed milk, blots were incubated with primary monoclonal antibodies specific for HCV E2 and cellular GAPDH (Santa Cruz Biotechnology Inc, USA) and secondary horseradish peroxidase-conjugated anti-goat and anti-mouse antibodies (Sigma Aldrich, USA). The protein expressions were evaluated using a chemiluminescence detection kit (Sigma Aldrich, USA).

### Statistical analysis

All statistical analysis was done using SPSS software (version 16.0, SPSS Inc). Data are presented as mean ± SD. Numerical data were analyzed using student's t-test and ANOVA. A *p *value < 0.05 was considered statistically significant.

## Results

### Relative expression analysis of HCV receptor genes in serum-infected Huh-7 cells

**Figure 1:**
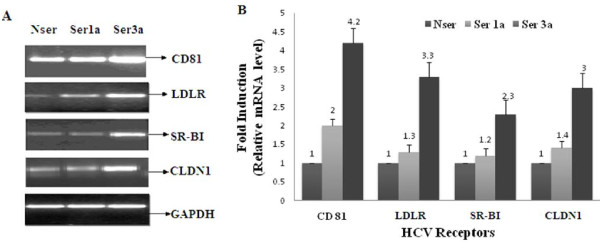
**Comparison of expression of CD81, LDLR, SR-BI and CLDN genes in HCV 3a and HCV 1a serum-infected Huh-7 cells**. **A) **Gene expression of CD81, LDLR, SR-BI and CLDN1genes in Huh-7 infected with HCV serum of genotype 3a (S3a) and HCV serum of genotype 1a (S1a) as compared to normal serum (NSer). Cells were harvested and relative RNA determinations were carried out using semi-quantitative PCR. **B) **Relative gene expression of CD81, LDLR, SR-BI and CLDN1genes in Huh-7 infected with HCV serum of genotype 3a (S3a) and HCV serum of genotype 1a (S1a) as compared to normal serum (N) by real time PCR. All experiments were performed three times independently with triplicate samples in each. Error bars indicate the mean plus or minus SD. *p < 0.01 vs. normal

In our previous studies we successfully established HCV serum infection in Huh-7 cells and monitored viral load [12,42,47]. In this study, we compared the mRNA expression of CD81, LDLR, SR-BI and CLDN1 genes in HCV genotype 1a and 3a serum-infected Huh-7 cells. HCV serum-infected liver cell lines as model cell culture system were used to study HCV entry receptors [7,12,48-51]. Expression of these HCV receptors was analyzed after total RNA isolation by semi-quantitative PCR and real-time PCR using gene-specific primers. Semi-quantitative results indicate the higher expression of CD81, LDLR, SR-BI and CLDN1 genes in Huh-7 cell infected with HCV genotype 3a serum as compare to genotype 1a (Figure 1A). Real-time PCR results indicate the up regulation of genes in HCV-3a serum infected Huh-7 cells as CD81 (4.2 fold), LDLR (3.3 fold), SR-BI (2.3 fold) and CLDN1 (3 fold) while in HCV-1a serum-infected Huh-7 cells the changes were: CD81 (2 fold), LDLR (1.3 fold), SR-BI (1.2 fold) and CLDN1 (1.4 fold) compared to normal serum (Figure 1B).

### Screening for siRNAs effective against HCV receptor CD81, LDLR, CLDN and SR-BI genes

siRNA-mediated RNAi is strictly sequence specific, so appropriately designed siRNAs targeting HCV genomic RNA can efficiently and specifically suppress HCV replication in vitro [52-54]. In vitro-transcribed sequence-specific siRNAs were designed against two regions of each HCV receptor i.e., siCD81, siCD81-B against CD81, siLDLR, siLDLR-B against LDLR, siSRBI, siSRBI-B against SRB1 and siCLDN1, siCLDNI-B against CLDN1 gene and scrambled (Sc) siRNA. Those which have been transcribed with nonspecific sequence have no homology to any known cellular genes. A scrambled sequence has been used to avoid any changes to the gene expression that may result from the siRNA delivery method. Scrambled Sc siRNA serves as a negative control (Table 1). Huh-7 cells were transfected with 100 nM of each of two siRNAs against each HCV receptor, then infected with HCV serum of genotype 3a for 48 hrs. Semi-quantitative PCR results showed that using siRNAs against HCV receptors CD81, LDLR, SR-BI and CLDN1 in serum-infected Huh-7 cells gave varied reductions in expression after 48 hr. The CD81 gene was maximally inhibited by siCD81-B, the LDLR gene by siLDLR, the SR-BI by siSRBI and the CLDN1 gene by both siCLDN1 and siCLDN1-B as compared to control (S3a) (data not shown). Therefore, in further experiments for silencing the expression of CD81, LDLR, SR-BI and CLDN1 genes we used only siCD81-B, siLDLR, siSRBI and siCLDN1 respectively.

**Table 1:** Sequences of siRNA used in this study

No	Name	Sequences
1	Scramble-antisense	AACCTGCATACGCGACTCGACCCTGTCTC
	Scramble-sense	AAGTCGAGTCGCGTATGCAGGCCTGTCTC
4	CD81 antisense	AAGTGCATCAAGTACCTGCTCCCTGTCTC
	CD81 sense	AAGAGCAGGTACTTGATGCACCCTGTCTC
5	CD81-B antisense	AAGATGCCTACATAGAAGGTGCCTGTCTC
	CD81-B sense	AACACCTTCTATGTAGGCATCCCTGTCTC
6	LDL antisense	AAATGCATCTCCTACAAGTGGCCTGTCTC
	LDL sense	AACCACTTGTAGGAGATGCATCCTGTCTC
7	LDL-B antisense	AACTCCCGCCAAGATCAAGAACCTGTCTC
	LDL-B sense	AATTCTTGATCTTGGCGGGAGCCTGTCTC
8	SR antisense	AAGCAACATCACCTTCAACAACCTGTCTC
	SR sense	AATTGTTGAAGGTGATGTTGCCCTGTCTC
9	SR-B antisense	AACATGATCAATGGAACTTCTCCTGTCTC
	SR-B sense	AAAGAAGTTCCATTGATCATGCCTGTCTC
10	CLD antisense	AATCTGAGCAGCACATTGCAACCTGTCTC
	CLD sense	AATTGCAATGTGCTGCTCAGACCTGTCTC
11	CLD-B antisense	AAGGCATTTGGCTGCTGTAAGCCTGTCTC
	CLD-B sense	AACTTACAGCAGCCAAATGCCCCTGTCTC

**Figure 2:**
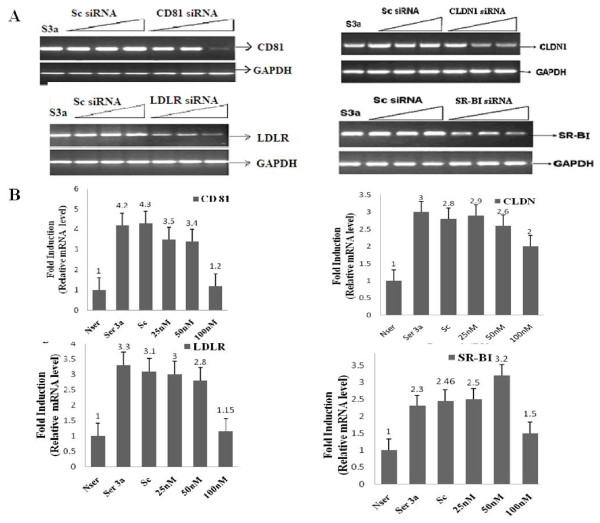
**Silencing of gene expression of HCV receptors by their specific siRNA in a dose dependent manner**. Huh-7 cells were infected with HCV-3a serum (S3a) along with or without 25 nM, 50 nM and 100 nM of siRNAs against HCV receptors CD81, LDLR, CLDN1 and SR-BI and scrambled (Sc) siRNA for 48 hrs. The scrambled (Sc) siRNA has nonspecific sequence with no homology to any known cellular genes. **A) **Semi-quantitative PCR analysis of gene expression of CD81, LDLR, CLDN1 and SR-BI using serial doses (25 nM, 50 nM and 100 nM) of scrambled siRNA or the specific siRNA siCD81, siCLDN, siLDLR, or siSR-BI respectively. **B) **Real-time PCR analysis indicating fold reduction of CD81, CLDN1, LDLR, and SR-BI gene using serial doses (25 nM, 50 nM and 100 nM) of scrambled siRNA or specific siRNAs of siCD81, siCLDN, siLDLR or, siSR-BI respectively.

Huh-7 cells were infected with HCV serum of genotype 3a (S3a) and treated with or without 25 nM, 50 nM and 100 nM of siRNAs against HCV receptors for 48 hrs post-transfection. Transient transfection of HCV receptor CD81-B, LDLR, SRBI and CLDN1 siRNAs in Huh-7 cells showed different effects on receptor RNA expression levels in a dose-dependent manner; but there was an optimal dose which showed maximum inhibition for all receptors with their specific siRNA. Results of semi-quantitative PCR indicate that expression of the CD81, LDLR CLDN1, and SRB1 receptor genes was significantly reduced at 100 nm siRNA as compare to scrambled siRNA, which showed no inhibition. Furthermore, real-time PCR results confirmed significant inhibition of mRNA expression of receptors CD81 (3-fold), LDLR gene (2-fold), CLDN1 (1-fold)and SR-B1 (0.8-fold) by using 100nM dose of siRNA against them as compare to HCV 3a serum infected Huh-7 cells without siRNA (Figure 2). The results of these dose-dependent experiments show that the optimal dose of siRNA which shows best inhibition of receptors is 100 nM for siCD81-B, siLDLR, siSRBI and siCLDN1. Using the results of this experiment, we screened the siRNAs against HCV receptors and selected the optimal dose of siRNA for further experiments.

### Silencing effect of HCV receptors siRNAs against HCV

**Figure 3:**
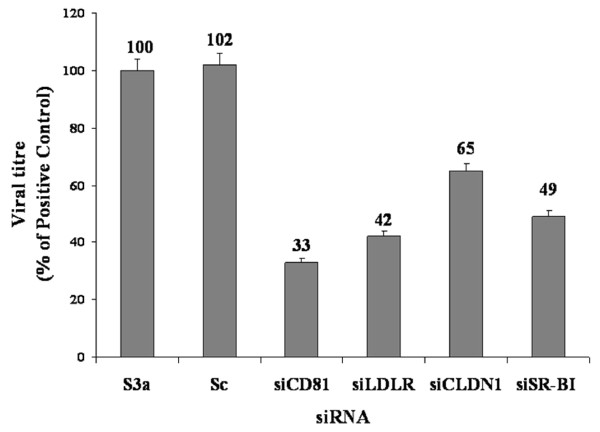
**Effect of silencing of HCV receptors by their specific siRNA on viral titer in Huh-7 cells**. **A) **Viral titer in Huh-7 cells treated with scrambled siRNAs (Sc) or siRNAs of CD-81, LDL, SR and CLD and incubated for 6 hrs before adding HCV-3a sera (Ser 3a). HCV RNA levels were quantified by real-time PCR. Data are expressed as mean percent viral load of non-siRNA treated samples. Three independent experiments with triplicate determinations were performed. Error bars indicate, means plus or minus SD. *p < 0.01 vs. Ser3a.

The cellular genes CD81, LDLR, SR-BI and CLDN1 that are functionally involved in HCV entry can also serve as potential targets for RNAi. Several studies have shown that siRNA against CD81 distinctly inhibited HCV entry (> 90%) in HCV serum infection irrespective of HCV genotype, viral load, or liver donor [55]. Furthermore, 90% down-regulation of SR-BI expression was also seen in Huh-7 cells by RNAi which caused a 30%-90% inhibition of HCVpp infection [56,57]. Silencing of CLDN1 also inhibited HCV infection in susceptible cells (Huh7.5) [58]. In the present study, we observed that sequence-specific siRNAs against the CD81, LDLR, SRBI and CLDN1 receptors significantly inhibit the expression of their respective genes. Keeping all this in view, we used in vitro-transcribed siRNA against all HCV receptors CD81, SR-BI, LDLR, CLDN1 and observed the effect of silencing of these receptors on viral titer. In the first step, we analyzed the effect on viral titer by silencing each receptor individually and in combination using siRNA against two receptors simultaneously. To determine whether siRNA against each HCV receptor can reduce viral load in HCV-infected cells, Huh-7 cells were infected with HCV serum with and without individual siRNAs (100 nM) against each HCV receptor, CD81, LDLR, SR-BI and CLDN1, for 48 hrs. Their RNA and viral loads were quantified by real-time PCR. Results showed a 67%, 58%, 35%, and 51% decrease in viral load incubated with HCV receptor CD81, LDLR, CLDN1 and SR-BI siRNAs, respectively compared to control (S3a), whereas no inhibition was observed with scrambled control siRNA (Figure 3).

**Figure 4:**
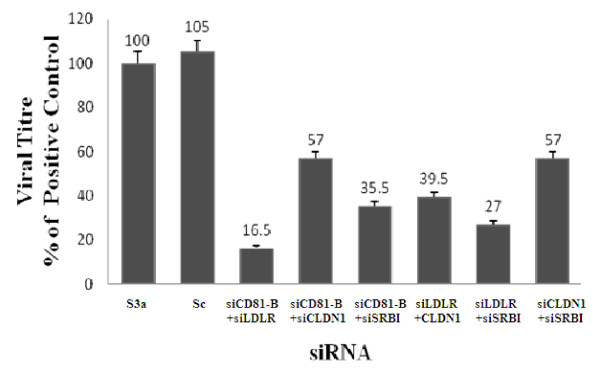
**Combined effect of specific siRNA silencing of HCV receptors on viral titer in Huh-7 cells**. Huh-7 cells were treated with scrambled siRNAs (Sc) or combinations of siRNAs (siCD81-B+siLDLR, siCD81+siCLDN1, siCD81+siSR-BI, siLDLR+ siCLDN1, siLDLR+ siSR-BI, siCLDN1+siSR-BI) and incubated for 6 hrs before adding HCV-3a sera (Ser 3a). HCV RNA levels were quantified by real-time PCR. Data are expressed as mean percent viral load of non-siRNA treated samples. Three independent experiments with triplicate determinations were performed. Error bars indicate indicate means plus or minus SD. *p < 0.01vs. Ser3a.

In the second step, to determine whether a combination of siRNA against respective HCV receptors can reduce viral load in HCV-infected cells, Huh-7 cells were infected with HCV serum with or without the following combinations of siRNA at 100nM: CD-81+LDLR, CD-81+SR-BI, CD-81+CLDN, LDLR+ SR-BI, LDLR+ CLDN, CLDN+SR-BI. At 48 hrs after treatment, the RNA and viral loads were quantified by real-time PCR. Results showed 83.5%, 43%, 64.5%, 60%, 73% and 43% decrease in viral load incubated with siRNA of CD-81+LDLR, CD-81+CLDN, CD-81+SR-BI, LDLR+ CLDN, LDLR+ SR-BI, CLDN+SR-BI, respectively as compare to control (S3a), whereas no inhibition was observed with scrambled siRNA (Figure 4). The siRNA combinations of siCD81 + siLDLR and siLDLR+ siSR-BI showed maximum inhibition of viral load.

**Figure 5:**
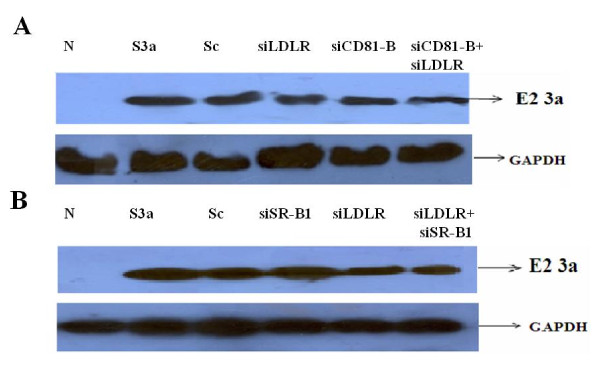
**Analysis of HCV E2 protein expression by using siRNA alone and in combination against HCV receptor genes CD81, LDLR and SR-B1**. Protein was isolated from Huh-7 cells treated with single or combinations of siRNA (siCD81-B, siLDLR and siSR-B1) against HCV receptor CD81 LDLR and SR-B1 genes and incubated for 6 hrs before adding HCV-3a sera (Ser 3a) for 48 hrs. Protein levels were quantified by western blot analysis using antibodies specific for E2 and GAPDH. **A) **Silencing of CD-81 and LDLR genes alone or in combination using specific siRNAs (siCD81-B, siLDLR) reduced HCV E2 protein expression levels in Huh-7 cells. **B) **Silencing of the LDLR gene or SR-B1 gene alone and in combination, using specific siRNA (siLDLR, siSR-BI, siCLDN1+siSR-BI) reduced HCV E2 protein expression in Huh-7 cells. GAPDH protein levels are also shown as an internal control and scramble siRNA (Sc) as siRNA control.

Furthermore, the effect of inhibition of HCV receptor genes CD81, LDLR and SR-B1 on the expression of viral proteins was determined by western blot analysis using specific antibodies. Huh-7 cell lysates infected with HCV serum of genotype 3a with or without siRNAs (100 nM each) against HCV receptors CD81, LDLR and SR-B1 for 48 hrs were separated by SDS PAGE and immunoblotted with antibodies specific for proteins. Results indicate a significant inhibition of expression of E2 3a, when a combination of siRNA (siLDLR+siCD81) were used as compare to individual siRNA against CD81 and LDLR (Figure 5). These results show the reduced total cellular viral protein expression due to the low expression of HCV envelop protein. Similarly, western blotting results indicate the significant inhibition of expression of LDLR and SR-B1 when a combination of siRNAs (SR-B1 +siLDLR) was used compared to individual siRNAs against LDLR and SR-B1 (Figure 5). This also shows the reduced total cellular viral protein expression due to the low expression of HCV envelops protein as well as LDLR receptor protein. The siRNA combinations of siCD81 + siLDLR and siLDLR+ siSR-BI showed maximum inhibition of viral envelope protein E2, which confirms the inhibition of HCV infection seen with siRNAs against HCV receptors

## Discussion

HCV entry into hepatocytes is the first step in the virus life cycle that results in productive viral infection, providing a major target for immunopreventive and therapeutic strategies [59-61]. Viral entry is thought to be mediated by HCV envelop glycoproteins E1 and E2 and several cell surface receptors which facilitate the binding of virus to host cells; but none of these cell surface factors alone are responsible for promoting HCV entry. Therefore, the interaction of HCV and its target host cells leading to the internalization of virus is considered a multistep process. These cell surface receptors mainly include the tetraspanin proteins CD81, SR-BI, and LDLR and the tight junction protein CLDN1 [62-66]. In this study, we have targeted HCV host cell surface receptors that interact with HCV structural genes causing HCV infection. Moreover, we analyzed the effect of siRNAs separately and in combination against HCV receptors on viral entry by quantifying the viral titer in siRNA-treated and non-treated serum-infected Huh-7 cells.

HCV envelop protein E2 posses glycosylation sites which interact directly with the cell surface receptors CD81, SR-BI and CLDN1 [67-69], confirming their role in HCV entry by using HCVpp or HCVcc infection in liver cell lines [70-73]. Nevertheless, the cell entry properties of HCVpp and HCVcc are different from those of serum-derived HCV because they are not associated with lipoproteins as is HCV naturally present in serum and do not employ LDLR for their entry into the cell [74-76]. These experimental models do not mimic the natural infection process, whereas recent investigations using serum-derived HCV (HCVser) infection of the human hepatoma cell line (Huh-7) are consider to recapitulate the in vivo situation as closely as possible [77]. We have described successful HCV serum infection in liver cells in our earlier studies [12,78,79]. In this study, we again utilized the serum-infected Huh-7 cell culture model to evaluate the comparative gene expression levels of CD81, SR-BI, LDLR and CLDN1 receptors induced by HCV serum of genotype 1a and 3a. Our results showed relatively high expression of CD81, SR-BI, LDLR and CLDN1 receptors in HCV serum-infected Huh-7 cells of HCV genotype 3a as compared to genotype 1a (Figure 1). This information revealed that active infection by serum-derived HCV particles in Huh-7 cells contributed to increased expression levels of these cell surface receptors during infection. Moreover, comparative studies of HCV 1a versus 3a serum infection of human hepatocytes suggests that the nature of cell-virion genotype combination is also a determinant factor for virus entry as both HCV genotypes induced different expression levels of cell surface receptors.

RNAi provides an exciting new technology that promises to be useful in treatment of viral diseases. Previously it has been reported that cellular genes functionally involved in HCV entry like CD81, LDLR, SR-BI and CLDN1 also serve as potential targets for RNAi. Several reports showed that potent RNAi against HCV gene activity reduced the serum infection 30%-90% [80-82]. Previously, we have shown the inhibition of HCV replication and HCV infection in liver cell lines by using siRNA against HCV structural genes Core, Envelop E1, E2 and cellular gene Cox-2 in liver cells [12,42,83]. In the current project, we utilized a similar RNAi strategy to silence the expression of HCV receptor genes to block the HCV entry in a serum-derived HCV-infected Huh-7 cell culture model and analyzedits effect on viral load. Assessment of the optimum dose regime of siRNA is essential to enhance the inhibition of target genes and to better make use of the knockdown mechanism while limiting off target effects [84]. Serial doses (25 nM, 50 nM, 100 nM) of siRNA transfected into Huh-7 cells were used with subsequent HCV 3a serum infection (Figure 2). Initially, two regions of each receptor were selected, and among these the most effective ones were transfected (100 nM) into Huh-7 cells infected with HCV serum of genotype 3a for further analysis. The HCV infection pathway employs enhancement in expression of cell surface receptors that may facilitate entry and increase viral load during infection.

In experiments to knock down the expression of host cell surface HCV receptors on Huh-7 cells using siRNA to block HCV entry against each receptor gene separately and in combination prior to infection with HCV serum of genotype 3a, the viral titer was detected by real-time PCR using primers against the 5 UTR of viral copies in cells from the fifth day post infection. Our results indicate a significant decrease in HCV viral load by 67% and 58% due to the silencing of HCV receptor CD81 (33 fold) and LDLR (42 fold) respectively when compared to control (S3a), whereas siSRBI and siCLDN1 showed comparatively less inhibition of viral load (Figure 3). Furthermore, HCV viral load was significantly decreased up to 84% with combinations of siRNAs due to the silencing of siCD81+siLDLR gene and siLDLR+SRBI as compared to other combinations of siRNA (Figure 4). These observations confirmed that CD81 and LDLR are putative receptors facilitating HCV infection cooperatively, which may play different roles during the course of infection. Moreover, different studies exhibit the feasibility of targeting host cell factors involved in infection, as they are not prone to mutations, as potential targets for siRNA therapy. Henry and colleagues [85], targeted the IRES, NS5B, and host cell receptor CD81 by using a triple shRNA expression vector which concurrently reduced the HCV replication, CD81 expression, and E2 binding. Targeting multiple sites of the HCV genome and host factors involved in HCV infection are a realistic and valid approach aimed at preventing the virus from developing resistance.

In summary, our data show that siRNAs specific for HCV cellular receptors not only reduce the receptor gene expression but also reduce viral titer and viral protein E2 in siRNA treated cells confirming their role in HCV infection. A combination of these siRNA (siCD81-B, siLDLR, SR-BI) showed an even more dramatic reduction of HCV entry. In light of the present results, we propose that the use of siRNAs to inhibit expression of HCV receptor proteins separately or in combination could be helpful in reducing HCV entry.

## Abbreviations

HCV: Hepatitis C Virus; siRNA: small interfering RNA.

## Competing interests

The authors declare that they have no competing interests.

## Authors' contributions

JS and KS designed the study, analyze the data and wrote paper. JS, KS, SB, IB, KM performed all lab work. SMH and AW helped JS and SB in data analysis and literature review and arranges data. HS was principle investigator of the study. All authors read and approved the final manuscript.

## Authors' information

Jahan S and Khaliq S (PhD Molecular Biology), Sumreen B (Mphil Molecular Biology), Khan M (Mphil Molecular Biology) and Siddiqui MH (Mphil Molecular Biology) are research scholars at CEMB. Ijaz B (MPhil Molecular Biology) and Ahmad W (MPhil Chemistry) are research officers at CEMB. Hassan S (PhD Molecular Biology) is head of Applied and Functional genomics lab, CEMB, University of the Punjab, Lahore.
